# Supplementary material for: The interactome of intact mitochondria by cross-linking mass spectrometry provides evidence for coexisting respiratory supercomplexes
Source: Mol Cell Proteomics. 2017 Dec 8;17(2):216–32. doi: 10.1074/mcp.RA117.000470 (PMC5795388; doi:10.1074/mcp.RA117.000470)
Supplement: Supplemental Data [file supp_RA117.000470_133922_0_supp_23978_fzffwf.zip › spectra_annotation/mito_DR_spectra_annotation/308-1-83-1-13-1.pdf]

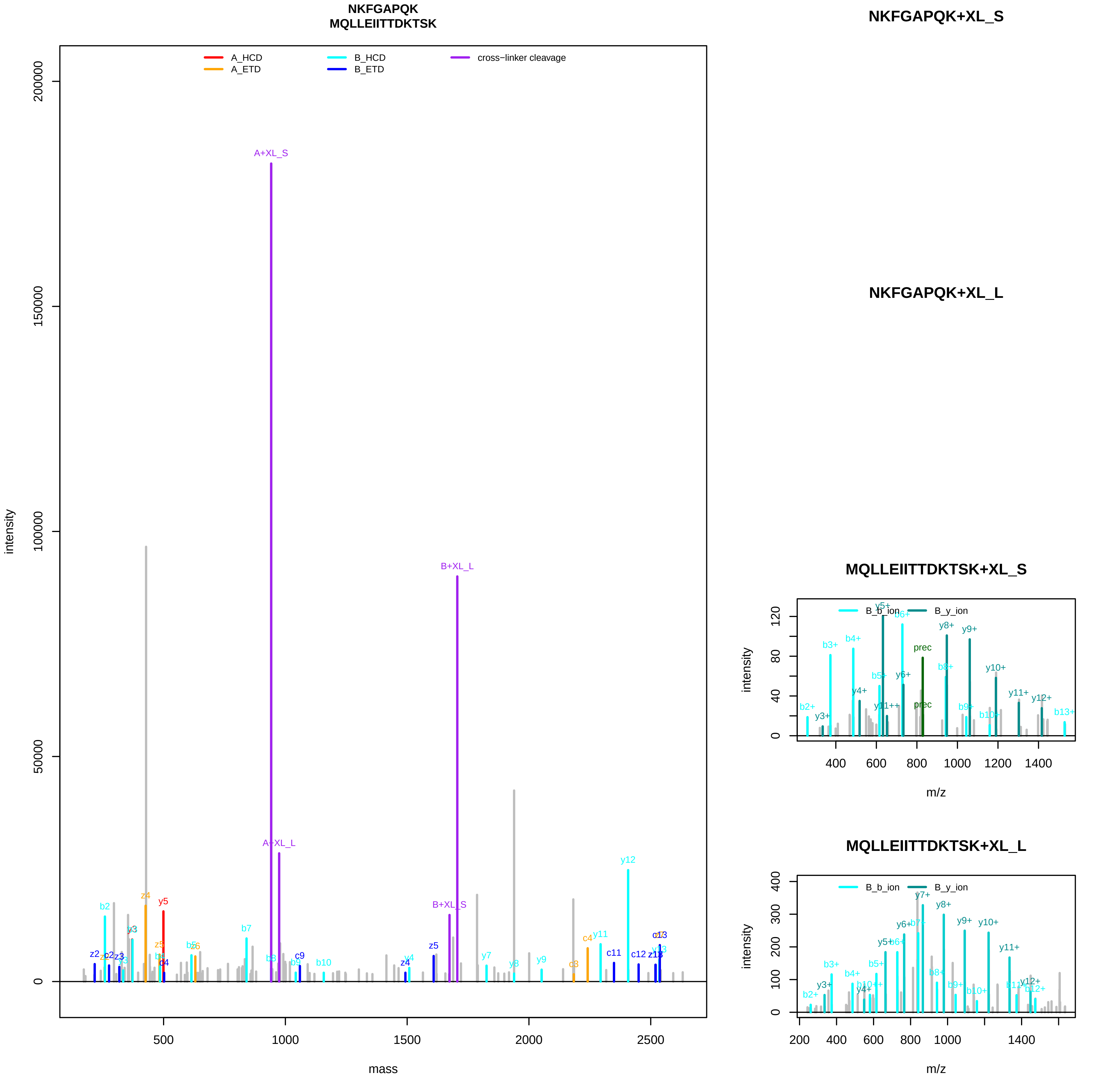
NKFGAPQK
MQLLEIITTDKTSK
A_HCD
A_ETD
B_HCD
B_ETD
cross−linker cleavage
intensity
mass
0
50000
100000
150000
200000
500
1000
1500
2000
2500
A+XL_S
A+XL_L
B+XL_L
B+XL_S
b2
y3
z4
y5
b7
y12
y11
c4
c11
c12
c9
z5
z4
y7
y9
b10
NKFGAPQK+XL_S
NKFGAPQK+XL_L
MQLLEIITTDKTSK+XL_S
MQLLEIITTDKTSK+XL_L
B_b_ion
B_y_ion
prec
m/z
0
40
80
120
400
600
800
1000
1200
1400
0
100
200
300
400
200
